# Histological Basis of Laminar MRI Patterns in High Resolution Images of Fixed Human Auditory Cortex

**DOI:** 10.3389/fnins.2016.00455

**Published:** 2016-10-07

**Authors:** Mark N. Wallace, Matthew J. Cronin, Richard W. Bowtell, Ian S. Scott, Alan R. Palmer, Penny A. Gowland

**Affiliations:** ^1^Medical Research Council Institute of Hearing Research, University of NottinghamNottingham, UK; ^2^Sir Peter Mansfield Imaging Centre, School of Physics and Astronomy, University of NottinghamNottingham, UK; ^3^Neuropathology Laboratory, Nottingham University Hospitals NHS Trust, Queen's Medical CentreNottingham, UK

**Keywords:** auditory cortex, myelin, magnetic resonance imaging, iron, myeloarchitectonics

## Abstract

Functional magnetic resonance imaging (fMRI) studies of the auditory region of the temporal lobe would benefit from the availability of image contrast that allowed direct identification of the primary auditory cortex, as this region cannot be accurately located using gyral landmarks alone. Previous work has suggested that the primary area can be identified in magnetic resonance (MR) images because of its relatively high myelin content. However, MR images are also affected by the iron content of the tissue and in this study we sought to confirm that different MR image contrasts did correlate with the myelin content in the gray matter and were not primarily affected by iron content as is the case in the primary visual and somatosensory areas. By imaging blocks of fixed post-mortem cortex in a 7 T scanner and then sectioning them for histological staining we sought to assess the relative contribution of myelin and iron to the gray matter contrast in the auditory region. Evaluating the image contrast in ${\text{T}}_{2}^{*}$-weighted images and quantitative ${\text{R}}_{2}^{*}$ maps showed a reasonably high correlation between the myelin density of the gray matter and the intensity of the MR images. The correlation with T_1_-weighted phase sensitive inversion recovery (PSIR) images was better than with the previous two image types, and there were clearly differentiated borders between adjacent cortical areas in these images. A significant amount of iron was present in the auditory region, but did not seem to contribute to the laminar pattern of the cortical gray matter in MR images. Similar levels of iron were present in the gray and white matter and although iron was present in fibers within the gray matter, these fibers were fairly uniformly distributed across the cortex. Thus, we conclude that T_1_- and ${\text{T}}_{2}^{*}$-weighted imaging sequences do demonstrate the relatively high myelin levels that are characteristic of the deep layers in primary auditory cortex and allow it and some of the surrounding areas to be reliably distinguished.

## Introduction

Significant challenges arise when trying to relate high resolution fMRI images to a standard brain using a standard three-dimensional coordinate system (Morosan et al., 2001; Maldjian et al., 2003). Variations in the size of the brain, patterns of cortical gyri, and the relative position of structures, confound the representation across all brains of a single structure, especially for a small cortical area like the primary auditory area (Rademacher et al., 2001; Wasserthal et al., 2014). Thus, it has become more common to try and relate coordinates for functional activity to a corresponding high-resolution structural image of the same brain (Fischl et al., 1999; Walters et al., 2003; Sigalovsky et al., 2006). This works well for many subcortical regions especially if they are large or have a distinctive structure; however, it is much more challenging when trying to identify functional areas of the neocortex. Attempts have been made to relate structural images to cytoarchitectonic maps (Cohen-Adad et al., 2012) such as the classic map of Brodmann (Garey, 1994). However, standard T_1_- or ${\text{T}}_{2}^{*}$-weighted images are more closely related to the distribution of myelin or iron in the cortex than cellular distribution (Eickhoff et al., 2005; Geyer et al., 2011). Currently MRI cannot provide much useful information about the size, form, or even density of neurons that are the features used to classify cytoarchitectonic areas in the neocortex; although it can detect the more distinctive areas of the hippocampal region because of their unique arrangement of cell bodies and fibers (Augustinack et al., 2014).

Images obtained at ultra-high-field with a 7 T MRI scanner can provide sufficiently high resolution to allow parcellation of the cortex based on signal variations that putatively show differences in myelin density and its laminar distribution (Eickhoff et al., 2005; Duyn et al., 2007; Waehnert et al., 2016). This involves the use of quantitative MR parameters, such as relaxation times, that are more directly related to myelin levels than conventional weighted images. Another advantage of quantitative images is that they are less affected by experimental conditions such as inhomogeneous magnetic fields. However, the acquisition of high-resolution images brings its own problems and this has limited its usefulness (Waehnert et al., 2016). To date, ultra-high-field (7T) scanners are only used for research, but with their increasing prevalence there is a prospect that they could become a clinical tool. This would be particularly useful in studies of the auditory and language regions, where even being able to identify the primary auditory area would be a useful anchor point for looking at the relative positions of the surrounding secondary areas (Sigalovsky et al., 2006). However, there have been two problems with this approach. One is that although human myeloarchitectonics is a long-established field of study, much of the classic literature was published almost a 100 years ago, in German language journals that are difficult to access and interpret. This problem has recently been addressed by a number of anatomists including Nieuwenhuys and Zilles who have reinterpreted the classic literature (Nieuwenhuys, 2013) and have synthesized it to form new myeloarchitectonic maps of the human cerebral cortex that may be of use in neuroimaging studies (Nieuwenhuys et al., 2015; Zilles et al., 2015). The second problem is in how to interpret the MR images in relation to myelin staining. A recent study (Fukunaga et al., 2010) has shown that the laminar pattern shown by ${\text{R}}_{2}^{*}$ (= 1/${\text{T}}_{2}^{*}$)-weighted structural images of the primary visual cortex is at least partly due to a clear laminar pattern of iron content, mainly in the form of ferritin. The iron seemed to be the dominant factor in determining the image contrast, but within the gray matter the myelin and iron had a very similar distribution, with both forming a dense band corresponding to the stria of Gennari. In general, free iron and ferritin in the brain are primarily associated with oligodendrocytes; which are particularly dense in white matter and are responsible for forming the myelin sheath that covers many axons (Connor and Menzies, 1996). Not all oligodendrocytes contain the same levels of iron, and so it is important to examine other parts of the neocortex to determine whether other cortical areas also show a close correlation between ${\text{T}}_{2}^{*}$ (or its inverse, ${\text{R}}_{2}^{*}$) contrast and the density of myelinated fibers and/or the levels of iron. We were particularly interested in assessing the correlation between image contrast and myelin density in the auditory cortex as it has been suggested that the primary auditory area can be identified in structural MR images, because of its high myelin content (Dick et al., 2012; Wasserthal et al., 2014; De Martino et al., 2015).

In this study we compared the contrast in ${\text{T}}_{2}^{*}$-weighted gradient recalled echo (GRE), quantitative ${\text{R}}_{2}^{*}$, and T_1_-weighted phase sensitive inversion recovery (PSIR) images with histological staining for myelin and iron. Using both T_1_ and ${\text{T}}_{2}^{*}$ dependent images gives potentially more information for identifying distinct cortical areas than the use of either alone (Wasserthal et al., 2014). Imaging and staining were carried out on auditory regions of the temporal lobe including the primary auditory cortex and on sections from the second visual area (Brodmann area 18) in the occipital lobe.

## Materials and methods

**Figure 1:**
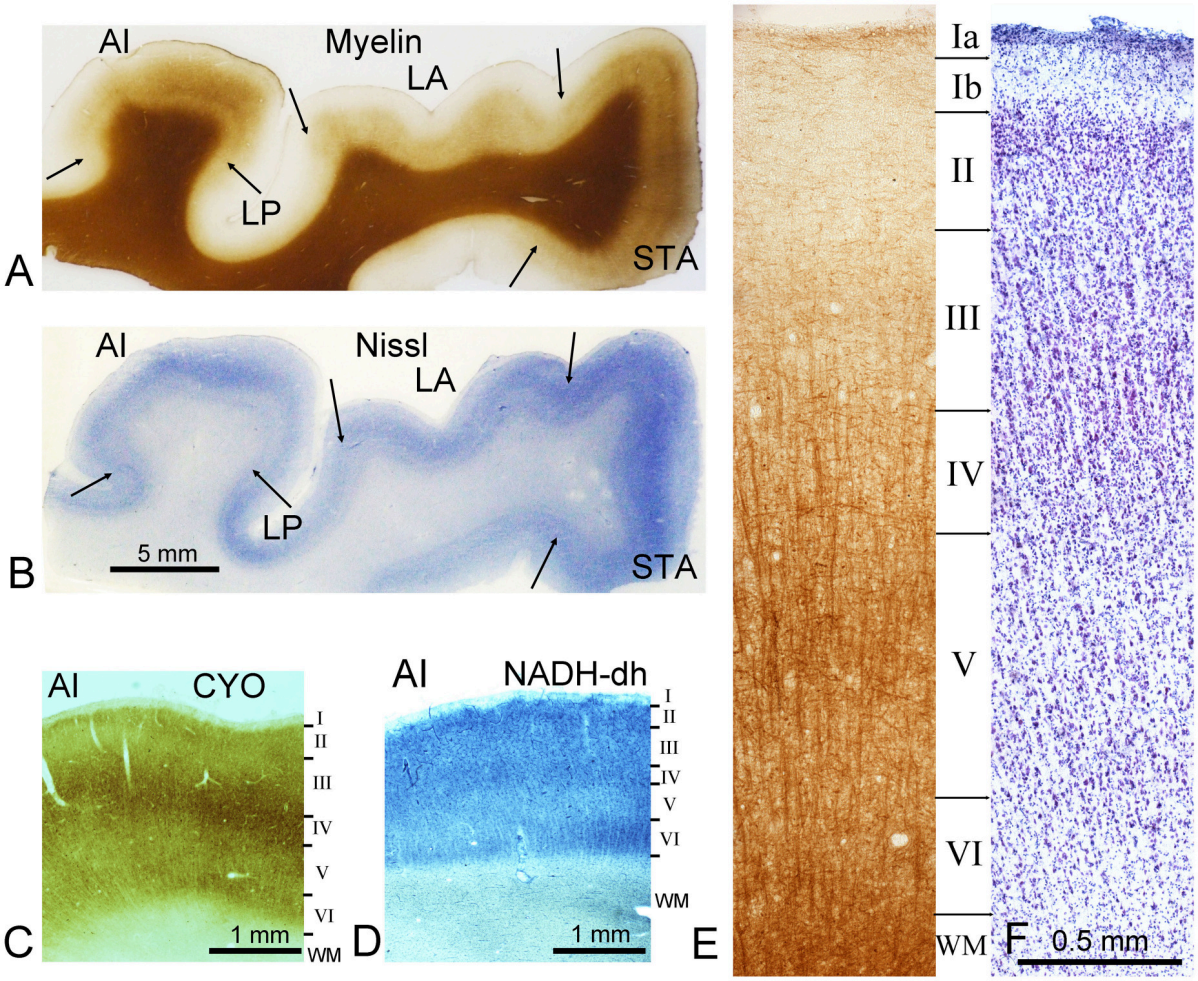
**Representative staining from the ventral bank of the lateral fissure**. Sections are at the level of the primary auditory cortex (AI) on the first transverse (Heschl's) gyrus. Sections were cut in the coronal plane. The sections in **(A)** and **(E)** are stained by the Gallyas silver stain for myelin while **(B)** and **(F)** are stained for Nissl substance. Section **(C)** was stained for cytochrome oxidase activity (CYO) while **(D)** was stained for NADH-diaphorase. As well as AI, several other areas can be identified in the first two panels and their borders are marked by arrows. These include the lateroposterior area (LP), the lateral area (LA), and the superior temporal area (STA). The later panels **(C–F)** all show sections through AI. The borders of cortical layers (defined by Nissl stain) are indicated by the horizontal lines and indicated by Roman numerals down to the white matter border (WM). This histological material was prepared for a previous study of the auditory cortex where the brain was fixed by perfusion through the internal carotid artery.

Samples of two human brains were obtained from the Nottingham Health Sciences Biobank using their generic ethical approval from the National Research Ethics Service. This study was carried out in accordance with the recommendations of the University of Nottingham Ethics committee and the tissue stored under the authority of the UK Human Tissue Act 2004. All subjects gave written informed consent in accordance with the Declaration of Helsinki. There were no significant neurological or pathological problems and the brains were considered normal for their age. The first was from a 58 year old man who died of sepsis following immunosuppression to allow treatment for multiple myeloma. The second was from an 81 year old man who died of bladder cancer and ischaemic heart disease. The brains were removed intact during post-mortem examination and fixed by suspension in 10% formalin for several months. The first brain was scanned in its entirety after rinsing it for 2 days in 0.9% saline to remove excess formalin. However, although the center of the brain was in focus, more lateral parts such as the auditory cortex were slightly blurred. The highest resolution images were all obtained from smaller blocks of tissue, that were removed from both brains and most of which were about 15 mm thick. These blocks were cut in approximately the coronal plane. They were rinsed for several days in 0.9% saline to wash out the excess formalin and then set in 1% agar by building up layers in a sphere of 20 cm diameter. Care was taken to minimize the formation of air bubbles either in the agar or at the interface with the brain. Other stained sections of human temporal lobe were available from a previous study of the auditory region where a variety of histochemical stains (see Figure 1) had been used on perfusion-fixed brain tissue (Wallace et al., 2002). In this study we have used the same definitions and nomenclature to identify cortical areas as that previous study, which also included the use of myelin in defining areas.

### MRI scanning

High resolution MR images were acquired using a Philips Achieva 7 T MRI system equipped with a 32-channel receiver head coil. T_1_- and ${\text{T}}_{2}^{*}$-weighted images were acquired, and quantitative ${\text{R}}_{2}^{*}$ (= 1/${\text{T}}_{2}^{*}$) maps generated in order to visualize variations in tissue composition across the cortex. For the ${\text{R}}_{2}^{*}$ values the log of the data was taken so that we could make a linear fit for each voxel through the echo times. Three-dimensional T_1_-weighted phase-sensitive inversion recovery (PSIR) images were acquired with 0.3 × 0.3 × 0.6 mm^3^ resolution (field of view = 25.2 × 105 × 105 mm^3^, flip angle = 26⋅, echo time = 4.05 ms, repetition time = 8.52 ms), with a scan duration of 6 h and 35 min. Images were acquired at two inversion times, using a tailored inversion pulse (Hurley et al., 2010) and then the PSIR image was calculated (Mougin et al., 2015). Multi-echo three dimensional ${\text{T}}_{2}^{*}$-weighted gradient-recalled echo (${\text{T}}_{2}^{*}$-wt. GRE) images were acquired with 0.3 × 0.3 × 0.3 mm^3^ resolution (field of view = 30 × 105 × 105 mm^3^, flip angle = 30⋅, echo time = 8/21/34 ms, repetition time = 200 ms), with a scan duration of 3 h and 55 min. Quantitative ${\text{R}}_{2}^{*}$ maps were calculated from the ${\text{T}}_{2}^{*}$-weighted images using MATLAB (The Mathworks, Inc., Natick, Massachusetts, USA).

### Histology

**Figure 2:**
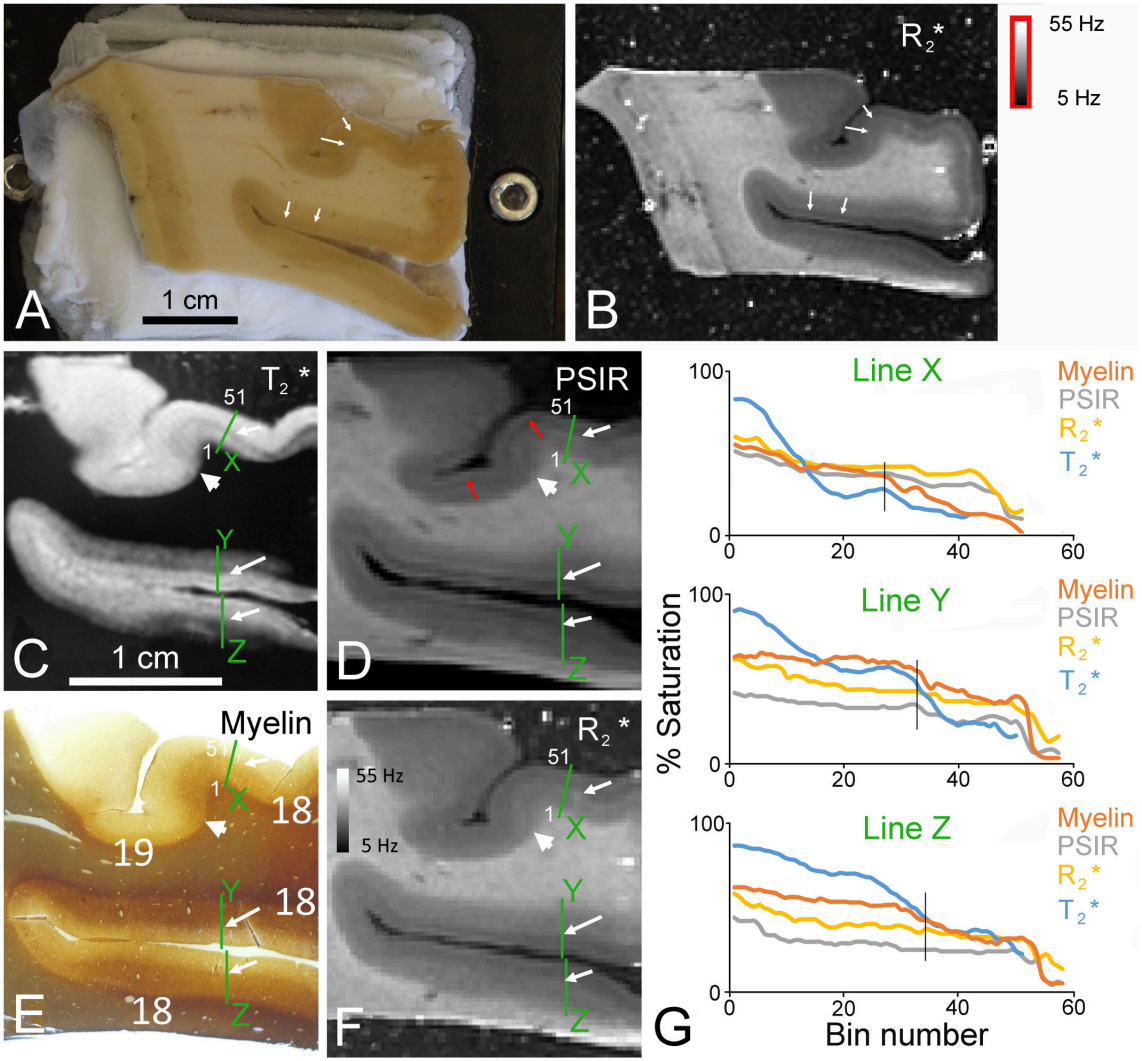
**Six images taken from approximately the same depth into a coronal block of the second visual area in the right occipital lobe from the first immersion-fixed brain**. Material from the same brain was also used to prepare the subsequent figures so that their fixation history was all the same. **(A)** Unstained block of brain photographed on the freezing microtome stage as it was being trimmed to a level that corresponded to that of an MR image. The white arrows point to a faint change in the color of the gray matter at the layer 3/4 border caused by layers 4–6 having more myelin and a creamier appearance. **(B)** Corresponding ${\text{R}}_{2}^{*}$ image of the same block shown in panel **(A)**. The corresponding line of transition between layers 3/4 is again shown by white arrows. In the myelin-stained section **(E)** the same border between the more deeply stained deep layers and the pale outer layers is indicated by the white arrows. The same junction is also marked by white arrows in the ${\text{T}}_{2}^{*}$-weighted GRE image **(C)**, T_1_-weighted PSIR image **(D)**, and quantitative ${\text{R}}_{2}^{*}$ map **(F)**. The border between Brodmann areas 18 and 19 is indicated by the large arrowhead in each of the four images **(C–F)**. The junction between a bright band present in the PSIR and ${\text{R}}_{2}^{*}$ images over layer 1 and the dark band in layer 2 is indicated by red arrows. This dual banding in layers 1/2 is not present in the myelin-stained section. Densitometric profiles measured across each of the green lines labeled *X* to *Z* are plotted as % saturation values as rolling means based on the surrounding 9 pixels (bin) and compared for each of the four images in panel **(G)**. The orientation of the profile is indicated for line X by indicating the position of the 1st and 51st bins in panels **(C–F)**. For all three profiles the line starts in the white matter and ends at the junction with the agar. Each bin has a side of 107 μm and the sample points are 60 μm apart. The vertical black line in each graph indicates the position of the border between layers 3/4 in the myelin staining.

After being scanned, the blocks were post-fixed for a week in 4% paraformaldehyde in 0.1 M phosphate buffer (pH 7.4) and then placed in 30% sucrose in the same buffer for 2 days or until they sank. They were then frozen and stored in a −80⋅C freezer before being sectioned at 40 μm on a sledge microtome with a freezing stage. The MR images were generally obtained from near the middle of the block, parallel to the surface cut in the coronal plane. The block was sectioned until the gray matter morphology corresponded with that shown in the MR image. An example of this is shown in Figures 2A,B. As the brain had not been perfused the gray matter is a more yellow color than the white matter due to the presence of many red blood corpuscles, but the contrast is reduced by the presence of many myelinated fibers in the gray matter, that can give it a creamier color. In some areas it is possible to identify the boundary between the deep myelinated layers of the cortex and the superficial layers that have low myelin levels (indicated by white arrows). Sections corresponding to the MRI image were mounted on Superfrost plus slides (Fisher Scientific) and then post-fixed for at least a day in 4% paraformaldehyde without any buffer. For the Gallyas (1979) myelin stain, sections were then dehydrated in a series of graded alcohols and immersed for 30 min in a solution of 150 ml pyridine and 75 ml glacial acetic acid. They were then rinsed three times in distilled water (no more than 5 min in each rinse) before pre-incubation for 1 h in a solution of 200 mg ammonium nitrate, 200 ml distilled water, 200 mg silver nitrate, and 1 ml of 4% sodium hydroxide (stirred vigorously and in that order). After three rinses in 0.5% acetic acid (3 min. each) they were placed in the physical developer made up of three stock solutions: (A) 25 g of anhydrous sodium carbonate in 500 ml of distilled water; (B) 400 mg ammonium nitrate, 400 mg silver nitrate, 2 g silicotungstic acid dissolved in 200 ml of distilled water; (C) 1 g ammonium nitrate, 1 g silver nitrate, 5 g silicotungstic acid dissolved in 36.5 ml of 4% paraformaldehyde (without buffer) in 463.5 ml of distilled water. The developer was made up by combining 100 ml of A, 20 ml of B, and 80 ml of C while stirring vigorously. Sections were then rinsed once in 0.5% acetic acid followed by three quick rinses in distilled water. The background was cleared by placing them in 0.2% potassium ferricyanide and fixed in 0.5% sodium thiosulphate (1 min.) before rinsing in distilled water. Sections were air dried, dehydrated in alcohols and mounted in Entellan mounting medium (Merck Millipore).

The presence of free iron (iron that is not bound to hemoglobin) was demonstrated by the diaminobenzidine intensification of Perl's reaction (Smith et al., 1997). Most free iron should be present as the ferric ion and can be revealed by potassium ferrocyanide, but we also checked for the presence of ferrous iron using potassium ferricyanide. Sections were post-fixed in 4% paraformaldehyde and mounted on gelatin-subbed slides before being air dried. Some were placed in a 1:1 mixture of 4 g of potassium ferrocyanide in 40 ml distilled water mixed with 40 ml of 5% hydrochloric acid, while others were placed in a similar mixture where the ferrocyanide was replaced with ferricyanide. Sections were incubated for 2 h in these solutions at 37⋅C and then washed in phosphate buffer. They were then intensified using the diaminobenzidine reaction (Smith et al., 1997).

Histological sections that corresponded best to the MR images were photographed and the images converted to gray scale values for analysis in Adobe Photoshop. The histological and MR images were superimposed to check that they were in register. Densitometric measurements were made along two to four radial lines, on either side of the green lines placed on the image, to provide mean density profiles across the cortex at three or four corresponding sites on each image. Measurements were made at regular intervals of 60 μm using a 3 × 3 pixel window to smooth out inconsistencies caused by blood vessels or local inhomogeneities in the distribution of myelinated fibers. The corresponding MR images were resampled to the same spatial resolution as the histological image (28.3 pixels/mm). Similar measurements were made across a series of corresponding lines in the MR images so that the profiles from the MR and histological data could be correlated. Density was recorded as % saturation of either the black or white scales depending on whether the “white” matter was dark or light in the image.

## Results

The thickness of the human neocortex is very variable depending on whether it is a primary sensory area on the surface of a gyrus (>3 mm for primary auditory cortex) or a secondary area in the depths of a sulcus (e.g., area LP at < 2 mm) as illustrated in Figure 1 which shows the ventral bank of the lateral fissure. For large areas such as the second visual area (Brodmann area 18) there are variations in both the total thickness and the proportionate thicknesses of the layers that form it. Thus, it has proved to be very difficult to establish clear morphological criteria for defining a neocortical area. Various histochemical markers that have been used to aid in identifying cortical areas such as cytochrome oxidase or NADH diaphorase are concentrated in layer IV of the primary sensory areas, including AI (Figures 1C,D). In most cortical areas, it is difficult to identify clear borders between layers and they do not coincide when Nissl and myelin stained sections are compared. This is illustrated in Figures 1E,F. where the approximate location of the layers is indicated by horizontal black lines and by convention these borders are determined by the changes in the size and distribution of the Nissl stained cell bodies. Also by convention layers defined by Nissl staining are indicated by Roman numerals as in Figure 1, while layers defined by myelin density are numbered with Arabic numerals. The clearest border is between layer I with its very low density of neurons and layer II with its high density of small neurons. Layer II gradually blends into layer III as the density of small neurons decreases and there start to be more of the larger pyramidal neurons. The border with layer IV is again indistinct, but layer IV has a higher density of small cells than the adjacent layers. Layer V has more of the larger pyramidal neurons, while layer VI has fewer of the larger pyramidal cells and like layer V it has a low density of neurons overall. Even the border of layer VI with the white matter is indistinct, because although the density of neurons drops there are some neurons in the white matter and the junction is difficult to identify at high power magnification, as there are many small glial cells in all cortical areas that stain for Nissl substance and obscure the neuronal patterns.

Layers are even less distinct in the myelin stained sections (c.f. Figures 1E,F) and the layer borders that can be discerned do not coincide with those in the Nissl-stained sections. In the part of area AI shown in Figure 1, there is a narrow band of tangentially running myelinated fibers in the outer part of layer I (Ia). Otherwise the outer layers have a comparatively low density of myelin staining. The staining density begins to increase in layer IV where there starts to be more prominent bundles of radially-oriented fibers, which continue on down to the white matter. There are also more tangentially running fiber bundles, particularly in the middle of layer V (inner band of Baillarger). However, even the border with the white matter is difficult to discern; and appears more like a steeper gradient of increasing myelin density than a sharp transition.

There is a better correlation between the myelin staining and the MR images, but even here the correspondence is imperfect (Figure 2). In blocks of the occipital lobe in the second visual area (Brodmann area 18) there was a good correspondence between myelin staining and the ${\text{T}}_{2}^{*}$-weighted images, but they were never perfectly matched. There was a relatively high and uniform density of myelinated fibers in the deep layers of the area 18 gray matter with a clear border between the dark layer 4 and the pale layers 1–3 (see white arrows in Figure 2E). This allowed the detection of the area 18 border, where it was adjacent to a less myelinated part of Brodmann's area 19 (see large white arrowhead). This border was reflected in the ${\text{T}}_{2}^{*}$-weighted image where the deep layers of area 18 were darker and the light/dark border occurred at the same place in both images (white arrows in Figure 2C). The same borders were present in an inverted form in the PSIR images (Figure 2D) and ${\text{R}}_{2}^{*}$ maps (Figure 2F). The superficial layers had very little myelin staining and a corresponding bright band in the ${\text{T}}_{2}^{*}$-weighted image. The ${\text{R}}_{2}^{*}$ and PSIR data followed the inverse of this behavior with a dark band in the superficial layers. There was also a narrow band of higher brightness in layer I of the PSIR and ${\text{R}}_{2}^{*}$ images even although there was no corresponding increase in the density of myelinated fibers. The PSIR and ${\text{R}}_{2}^{*}$ images were very similar to each other and to the myelin values as shown by the similar intensity profiles at three lines across the cortex which have been plotted together in Figure 2G. These profiles start in the white matter and in most cases extend into the agar so that we could check how low the baseline measures were when there was no tissue. The exception was the ${\text{T}}_{2}^{*}$ image where the profile was cut short just before the edge of the cortex because the agar had a similar value to the white matter and so didn't provide a baseline.

**Figure 3:**
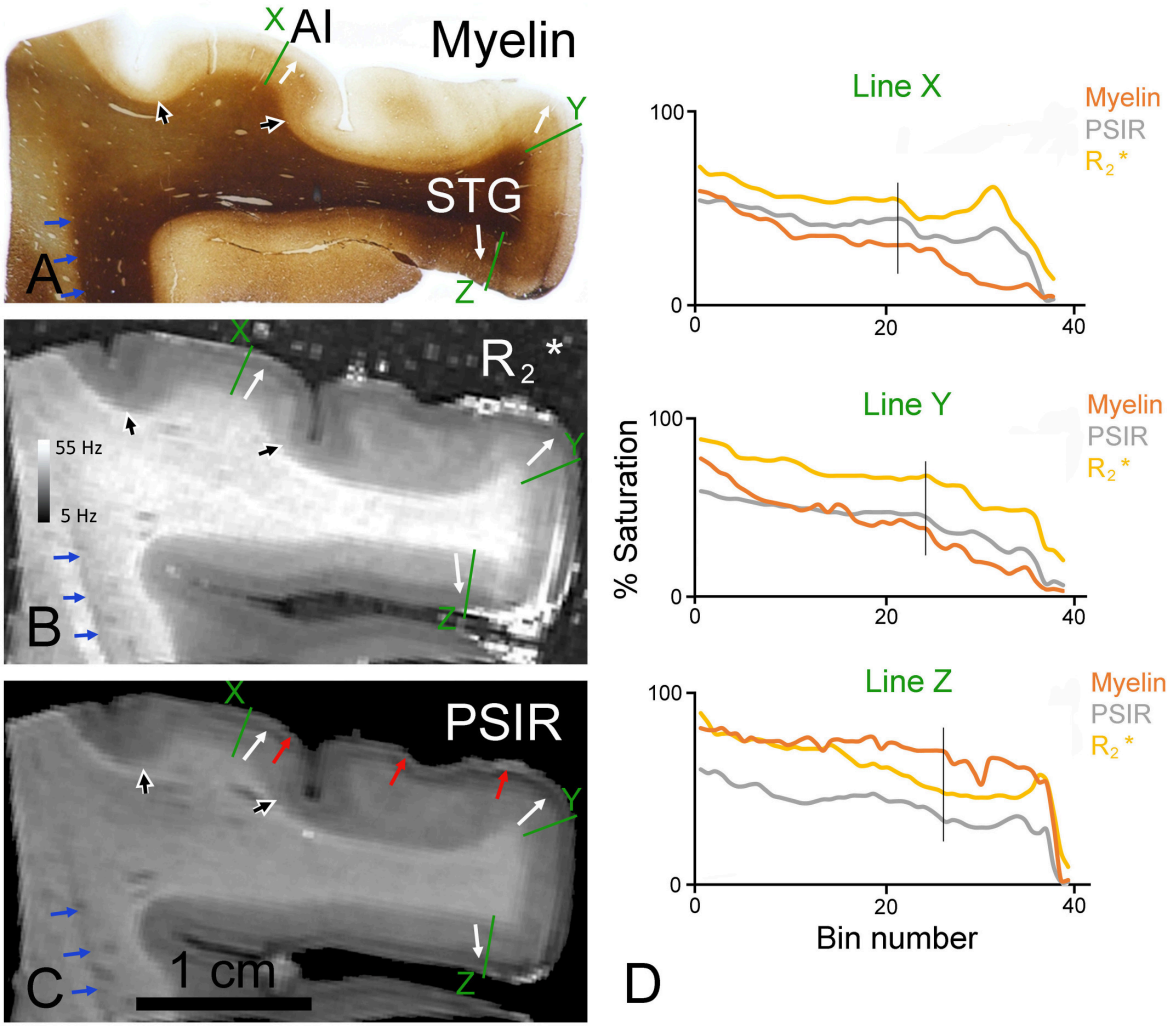
Coronal sections through the ventral bank of the lateral fissure stained for myelin (A) or viewed as quantitative ${\text{R}}_{2}^{*}$ (B) or PSIR images (C). The junction between the deep layers (4–6) with their greater density of myelinated fibers and the superficial layers (1–3) with their low density of myelinated fibers is indicated by white arrows in the primary auditory cortex (AI) and the superior temporal gyrus (STG) of all three images. The borders of AI are indicated by the large black/white arrowheads in all three images. The border between the bright band in layer I and the dark band in the layer II observed most clearly in PSIR images is marked by red arrows. The edge of a narrow dark band along the margin of the white/gray matter boundary at the edge of the insula is indicated by the blue arrows. The densitometric profiles across the green lines are plotted in **(D)** for the myelin and the two MR images with bin 1 being in the white matter and bin 36 close to the surface. The vertical black line in the graphs indicates the position of the 3/4 border in the myelin staining.

In blocks of the right temporal lobe that included the superior temporal gyrus and the first transverse gyrus (Heschl's gyrus) a similarly good correspondence was observed between the density of myelinated fibers and the ${\text{R}}_{2}^{*}$ values. In the myelin staining there was a moderate density of myelinated fibers in the deep layers and a relative lack of myelin in the upper layers (Figure 3A). This was the case both in the primary auditory cortex (AI) and on the superior temporal gyrus (STG). The pattern in the cortex between these two areas was distorted because the section was cut obliquely through an area of folded gyrus and the regular six-layered pattern of the cortex was no longer apparent. There were also some faint striations at the edge of this distorted area which were caused by the sectioning process. The thickness of layers 4–6 becomes sharply reduced at the borders of AI and this is marked by the black/white arrowheads in all three images (Figures 3A–C) There was a corresponding division in the MR images with the same border at the layer 3/4 junction manifesting as a light/dark transition (corresponding white arrows in the different panels of Figure 3). However, in the ${\text{R}}_{2}^{*}$ and especially the PSIR images there was also a prominent subdivision in the outer layers with a dark inner band roughly corresponding to layers 2/3 and a brighter outer band roughly corresponding to layer 1 that did not have a corresponding feature in the myelin staining (red arrows in Figure 3C). Another example of this black/white banding occurring at a junction is seen in Figure 3B where there are light and dark bands at the junction of the gray and white matter at the edge of the insula (blue arrows in Figure 3). The MRI light/dark transition corresponds to the gray/white matter transition in the myelin staining, but the narrow dark band in the MR images does not have any corresponding structure in the myelin staining. When the densitometric profiles are plotted for radial lines across the cortex the changes in the myelin, PSIR and ${\text{R}}_{2}^{*}$ images were found to be reasonably similar with correlation values of 0.76–0.95. The main differences were the result of artifacts in the ${\text{R}}_{2}^{*}$ images caused by bubbles in the surrounding agar or imperfections in the fixed tissue. Gradients in the fixation may also have caused slight changes in the darkness of myelin staining in the white matter which were not replicated in the MR images.

**Figure 4:**
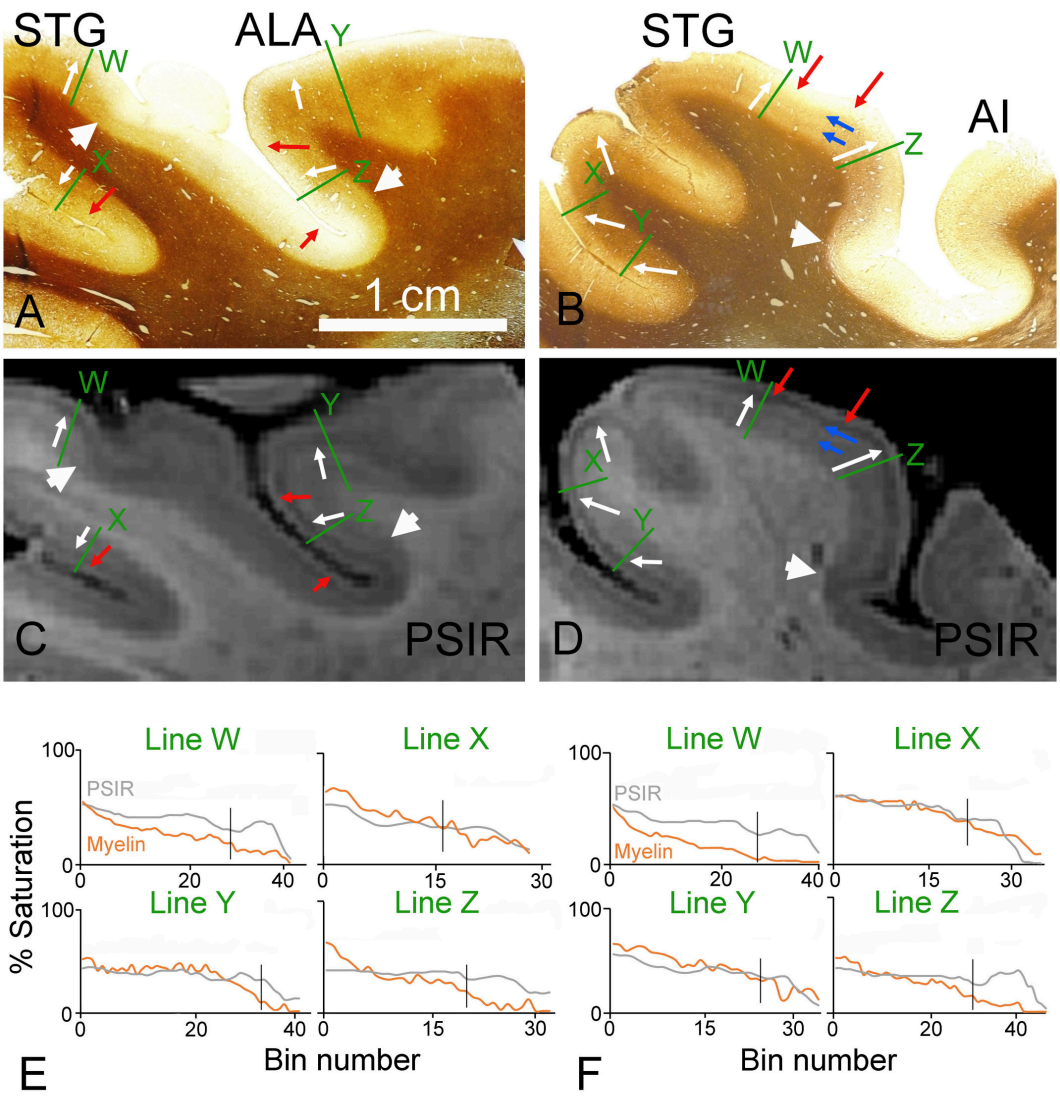
**Coronal sections through the ventral bank of the lateral fissure stained for myelin (A,B) or viewed as PSIR images (C,D)**. The sections in **(A,C)** cut through the area that is anterior and lateral (ALA) to the primary auditory part of the first transverse (Heschl's) gyrus as well as the superior temporal gyrus (STG). The sections in **(B,D)** are from further posterior on the STG. The junction between the well-myelinated deep layers (4–6) and the pale superficial layers is marked by white arrows. The borders between myeloarchitectonic areas are indicated by the large white arrowheads in the myelin sections and the corresponding density changes are also indicated in the PSIR images. The junction between the bright outermost band (layer I) and the dark band in layer II of the PSIR images is marked by red arrows and the corresponding points are indicated in the myelin sections. The blue arrows mark the faint inner and outer bands of Baillarger in the myelin staining of section **(B)** and the PSIR image of **(D)**. The densitometric profiles across the green lines are plotted in **(E,F)** for the myelin and PSIR images above them. The vertical black line in the graphs indicates the position of the 3/4 border in the myelin staining.

In a block from a more anterior position in the left temporal lobe a separate auditory area is located anterior and lateral to the primary auditory area (the anterior lateral area–ALA; (Wallace et al., 2002)). For this block and the next only PSIR images were produced because they seemed to show slightly higher contrast than the ${\text{R}}_{2}^{*}$ images and had the same patterns. The myelin staining in this area is also darker in the deep layers than in the pale outer layers and is similar to the pattern in the superior temporal gyrus (STG; Figure 4A). Although there are subtle changes in the myelin staining in the deep layers there is no evidence of any laminar pattern in the deep layers of either area. However, in the corresponding PSIR image (Figure 4C) there are four alternating bands of light/dark signal. The middle of these PSIR transitions corresponds to the light/dark myelin staining that can also be seen in the STG (see white arrows). The outermost light/dark PSIR transition does not have any corresponding change in myelin staining in any of the cortical areas (red arrows). Despite this confusing laminar pattern, it is still possible to discern the borders of a myeloarchitectonic area, with low levels of myelin staining in the deep layers, by examining the PSIR image (see large white arrowheads). In a block taken from a more posterior part of the left STG (Figure 4B), there is again a darker myelin staining in the deep layers and pale staining in the superficial layers (white arrows). This junction corresponds to a pale/dark junction in the PSIR image as well (Figure 4D). However, the PSIR image, once again, has a light/dark transition that is not present in the myelin staining (red arrows). Conversely, the myelin staining in the deep layers has a faint banded pattern with an inner and outer band of Baillarger (blue arrows). There is also a faint indication of a corresponding banding pattern in the PSIR image. The presence of an architectonic area at the margin of the STG is more clearly evident in both the myelin and PSIR images (large white arrowhead). When the densitometric profiles were compared at corresponding points across the cortex they were generally similar (Figures 4E,F). However, in both sets of images the profiles labeled “Z” had a marked deviation from each other, near the surface of the cortex, indicating that factors other than myelin density were also affecting the PSIR image. There was some indication of banding in the white matter of these MR sections but this mainly appeared to be related to the presence of large veins some of which were oriented in the plane of the section.

**Figure 5:**
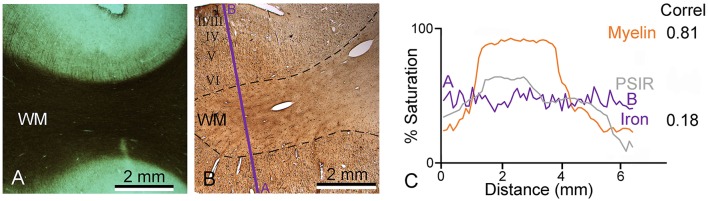
**Densitometric profiles were measured across nearby parts of the same gyrus to quantify myelin staining intensity (A), iron (B), and the PSIR image**. Measurements were made across a straight line indicated by the purple line in the direction from A to B and plotted in panel **(C)**. The correlation between the myelin and PSIR profiles was 0.81 while that between the PSIR and iron profiles was much lower (0.18).

**Figure 6:**
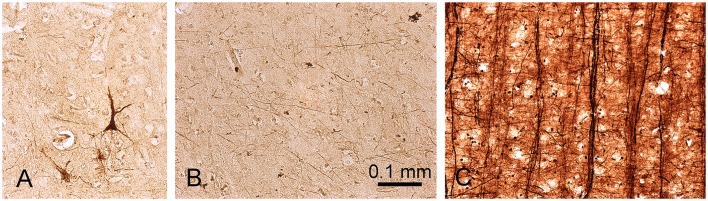
**Presence of ferric iron in sections from the planum temporale of the temporal lobe**. A few pyramidal neurons are stained for iron **(A)** but the majority of iron staining occurs in a loose network of fibers that occur in all cortical layers **(B)**. These fibers may be myelinated, but they do not correspond to the prominent bundles of myelinated fibers which form the output axons from pyramidal cells in this area and which have been stained with Gallyas's method in **(C)**.

In order to try and explain the partial mismatch between the myelin stain and the MR images, sections from the same blocks were also stained to determine the presence of iron. The iron staining was not associated with any laminar pattern in the areas of the temporal lobe that we examined. When a densitometric profile was measured by taking the mean of four parallel lines across a gyrus to compare the myelin staining, iron staining, and PSIR image, both the PSIR and myelin images had the densest peak in the while matter and a gradually falling density in the layers closest to the surface. This gave a correlation of 0.81 (Figure 5). However, the iron staining had a similar density across the gray and white matter and this gave a low correlation of 0.18 between the iron and PSIR profiles. The free iron was associated with a diffusely arranged network of fibers in all cortical layers (Figure 6B). There were small clumps of iron staining that may have corresponded to oligodendrocyte cell bodies and on rare occasions there was iron staining in a few isolated pyramidal cells (Figure 6A). The iron stained fibers may have corresponded to a subset of intrinsic axonal branches that were myelinated. However, they did not correspond to the main pyramidal cell output axons which were organized into prominent myelinated bundles in layers IV to VI (Figure 6C).

## Discussion

The longitudinal relaxation time, T_1_, and transverse relaxation time, ${\text{T}}_{2}^{*}$, of the MR signal in brain tissue are predominantly influenced by the iron content (Fukunaga et al., 2010), myelination (Lee et al., 2012), and degree of binding of water to macromolecules within each imaging voxel (Bock et al., 2009). Heme iron and calcium can also affect the local magnetic field, but these are less relevant for post-mortem tissue from healthy brains (Cohen-Adad, 2014) where most of the blood will have drained into the thorax and there shouldn't be any pathological calcification associated with blood vessels. Increases in iron, myelination and water binding change both T_1_ and ${\text{T}}_{2}^{*}$ by different degrees, leading to different contrast in each type of image. T_1_ values are more heavily influenced by myelination and water binding, and lower T_1_ values lead to hyperintensity in T_1_-weighted images. ${\text{T}}_{2}^{*}$ is more sensitive to iron content than T_1_, and lower ${\text{T}}_{2}^{*}$ values lead to hypointensity in ${\text{T}}_{2}^{*}$-weighted images. Quantitative ${\text{R}}_{2}^{*}$ maps show inverted contrast compared to ${\text{T}}_{2}^{*}$-weighted images and are particularly sensitive to iron concentration (Langkammer et al., 2010). While contrast in any weighted image is dependent on the imaging parameters as well as the relaxation parameters, contrast in quantitative ${\text{R}}_{2}^{*}$ maps is largely independent of the imaging parameters used. T_1_ and T_2_ relaxation parameters are reduced by formalin fixation (Dawe et al., 2009; Lee et al., 2012) and in this study we tried to minimize the role of fixation by partially reversing the cross-linking effects of the formalin before imaging the brain. The ${\text{T}}_{2}^{*}$ relaxation rate is also different for micro compartments that depend on the orientation of myelinated fibers relative to the main magnetic field (B_*o*_; Lee et al., 2011; Wharton and Bowtell, 2012; Sati et al., 2013). The effect of differences in fiber orientation has been shown to be much greater in fresh tissue than in fixed (Lee et al., 2012) and the use of post-mortem tissue meant that we could not easily assess the importance of bound water or fiber orientation in this study. Instead the results are more directly related to myelin and iron distribution.

One major concern of the current study is that the prominent banding pattern present in the outer layers of the cortex, particularly in the PSIR images, may be an edge artifact produced by a Gibbs overshoot in the inverse Fourier analysis used to produce the images. We had attempted to minimize the Gibbs truncation artifact by using relatively high sampling rates and reducing the field of view. However, it is not possible to remove the overshoot artifact entirely and the prominent light/dark bands in the outer one mm of the cortex (layers I–III) may not be reflecting any physical property of the tissue. This banding in layers I–III was most prominent in the PSIR images but was also faintly present in the ${\text{R}}_{2}^{*}$ images as well. The artifact may also have occurred at high contrast gray/white borders in the middle of the block at the edge of the insula (Figure 3B).

### Influence of iron on image contrast

The effect of iron distribution on ${\text{R}}_{2}^{*}$ images has already been studied in post-mortem visual and somatosensory cortex (Fukunaga et al., 2010) and we wanted to focus on the auditory cortex in this study. The primary visual cortex was an obvious region in which to study the relationship between MR contrast and myelin/iron content because of its distinctive band of dense myelinated fibers in layer IV. This feature makes it easy to identify reliably in whatever orientation it is sectioned (Sánchez-Panchuelo et al., 2012). However, it is the most atypical of neocortical areas because during primate evolution a new population of neurons became established in layer IV, that are not found in other cortical areas (Rockel et al., 1980). The distinctive stria of Gennari that distinguishes primary visual cortex contains a plexus of myelinated fibers running parallel to the cortical surface that is coexistent with the band of high ferritin content (Fukunaga et al., 2010). These myelinated fibers are not present in such high numbers in any other cortical area (Vogt and Vogt, 1919) and if the dense band of ferritin is also absent in other cortical areas then this would imply that the ferritin is mainly associated with the intrinsic axons of the recently evolved neurons in layer IV.

In the auditory cortex, we found a diffuse plexus of iron containing fibers but they did not form a distinct laminar pattern. When the density of iron staining was compared with the PSIR image intensity there was a low correlation. In the PSIR image there was a clear contrast between gray and white matter whereas the iron staining had similar levels in both. In the normal brain most non-heme iron is bound to ferritin and is present in the myelin sheath formed by oligodendrocytes as they require iron-dependent enzymes to produce and maintain myelin (Todorich et al., 2009). Some of the non-primary auditory areas are characterized by prominent bundles of myelinated fibers which are arranged in a regular pattern in the deep layers (Wallace and He, 2011; Zilles et al., 2015). These form distinctive radial bundles, but there was no sign of them in the sections stained for iron. Thus, in the auditory region, there was little correlation between the iron staining and the presence of myelin. In pathological brains, iron is also associated with β-amyloid plaques and nearby microglia (Smith et al., 1997; van Duijn et al., 2013), but we found no evidence of iron in microglia in the blocks examined. A few pyramidal cells were found to stain for iron and these probably indicate neurons where there has been a pathological accumulation of free iron linked to aging (Ward et al., 2014). Thus, although iron is present in the cortical gray matter of the auditory region, it may not contribute directly to the prominent laminar pattern present in the MR images. Nearly all the iron that we were able to demonstrate histologically was present in the ferric state and there was almost no evidence of the ferrous ion in levels that could be demonstrated histologically. This is partly because the DAB amplification method used to demonstrate the ferric ion does not amplify the signal from the ferrous ion, but also because there are very low levels of stainable ferrous ion in the normal brain (Smith et al., 1997).

We did not stain blocks of tissue for iron if they had already been used in producing the MR images. Formalin fixation interferes with the MR image and we had tried to wash out the excess formalin by washing for 2 days in saline before imaging. When we tried to stain these blocks for iron there was almost none detectable in the gray matter. It appeared that much of the free iron had been leached out of the cortex by the slightly acidic (pH 5) saline that was used for the washing. This type of leaching has been described previously (Smith et al., 1997). Washing in saline also affects the myelin staining as the staining method involves a process similar to photographic development and is affected by minute changes in the pH and redox state of the surrounding tissue. The myelin staining is also affected by ions such as the negatively charged phosphate ion that are normally used to buffer the pH levels. The phosphate binds to ionic silver and prevents it being reduced to metallic silver crystals. Thus, the best iron and myelin staining was obtained in blocks that had not been imaged beforehand.

### Relationship of myelin to image contrast

The PSIR images did display laminar patterns that looked similar to those seen in the corresponding myelin stained sections in both the primary area and some of the surrounding areas. This was also true of the myelin pattern and the ${\text{R}}_{2}^{*}$ maps and both the ${\text{R}}_{2}^{*}$ and the ${\text{T}}_{2}^{*}$w images had similar patterns to the myelin density. This supports the contention that MR images of the auditory cortex primarily register the levels of myelin and can be used for detecting differences between the degree of myelination in different auditory cortical areas as suggested previously (Sigalovsky et al., 2006; Lutti et al., 2014; Wasserthal et al., 2014). However, edge artifacts associated with the imaging methods used, mean that the appearance of banding within the outer cortical layers of the MR images do not represent a corresponding laminar pattern in the myelin of the gray matter.

Even at 7 T, MRI does not have adequate resolution to detect the thin bands of myelinated fibers which can be present in the neocortex and the images are subject to artifacts associated with post-mortem changes in the brain. A bright band roughly located over layer I of the cortex has already been shown in T2^*^ images of post-mortem brain and it has no relationship to the myelin staining (Geyer et al., 2011). Up to 14 sublayers have been described in myelin stained areas of the neocortex (Vogt and Vogt, 1919). These include four bands of dense myelinated fibers running parallel to the surface in layers 1a, 3a (Kaes-Bechterew), 4 and 5b (outer and inner bands of Baillarger). In this study, we achieved an isotropic resolution of 300 μm by exploiting the high sensitivity of 7T MRI and using a long data acquisition time. This is adequate to detect the enlarged outer band of Baillarger in layer 4 of the primary visual cortex, but may not be sufficient to detect the bands in other layers which can have a thickness of as little as 100 μm (Zilles et al., 2015). Another problem is the different compartments of water molecules associated with large macromolecules such as nuclear chromatin or the myelin sheath. There are thought to be two components to the T_1_ associated with the myelin sheath: a short component linked to the water contained in the lipid bilayers and a longer component linked to water in the axoplasm and extracellular space (Bock et al., 2009). However, fixation gradients and damage associated with placing the brain in formalin and then washing it in saline will lead to damage to the lipid bilayers and ultrastructure of the cells. This may have contributed to an artifactual banding pattern at the outer edge of the cortex even although the amount of myelin was preserved.

Imaging the brain *in vivo* also has problems due to pulsations associated with systolic blood pressure, breathing and the active flow of blood. Thus, we have not yet reached a position where we can use subtle differences in myeloarchitecture (Beck, 1928; Nieuwenhuys et al., 2015) in order to distinguish between the numerous belt areas that are present in the auditory cortex (Moerel et al., 2014; Leaver and Rauschecker, 2016). Despite this, *in vivo* structural MRI imaging is already being used to distinguish between cortical areas such as the primary auditory area with its high levels of myelin in the deep layers and adjacent areas such as LP which have much lower levels of myelin overall (Lutti et al., 2014). Identification of the primary auditory area, the position of which, varies considerably between different individuals (Rademacher et al., 2002; Da Costa et al., 2011) has been a valuable contribution. We have confirmed that the myelin density is a major contribution to MRI image contrast both in AI and some of the surrounding areas and should allow the identification of many areas in addition to primary sensory ones.

In future, even if the T_1_- and T_2_- weighted parameters are used together to produce an objective identification of areal borders, an *in vivo* myeloarchitectonic map by itself will not be sufficient to make a comprehensive identification of all the areas in the temporal lobe (Glasser and Van Essen, 2011; De Martino et al., 2015). Supplementary methods will be required that may include receptor binding studies (Morosan et al., 2005; Amunts et al., 2010) and the identification of the primary and additional areas by functional mapping (Moerel et al., 2014; Schönwiesner et al., 2015). Functional MRI should be able to reliably define at least two core auditory areas (Da Costa et al., 2011) and up to four adjacent belt areas by identifying discrete tonotopic maps (Moerel et al., 2014; Saenz and Langers, 2014). It should be possible to acquire these data before starting the main part of a study and combine it with the structural MRI data to make a more reliable map than would be available by either method alone (De Martino et al., 2015). However, in the meantime structural MR images alone should be sufficient to reliably detect the primary auditory area and some of the adjacent areas on the temporal lobe.

## Author contributions

The study was conceived and designed by MW, AP, IS, and PG. Brain samples and relevant permissions were collected by MW and IS and they prepared the blocks for histology. MC, PG, and RB designed and performed the imaging sequences for the blocks of brain and helped with interpreting the images. MC and MW analyzed the results and wrote the manuscript with the help of AP. All authors approved the final version.

## Funding

This work was funded by a core grant from the MRC supporting hearing research and a joint grant from the MRC and EPSRC on: Realizing the benefits of structural and functional MRI at ultra-high-field.

### Conflict of interest statement

The authors declare that the research was conducted in the absence of any commercial or financial relationships that could be construed as a potential conflict of interest.
